# A pain in the neck: *Salmonella spp.* as an unusual cause of a thyroid abscess. A case report and review of the literature

**DOI:** 10.1186/s12879-020-05161-w

**Published:** 2020-06-22

**Authors:** Maha K. AlYousef, Ahmed A. Al-Sayed, Ayham Al Afif, Uthman Alamoudi, Jaclyn M. LeBlanc, Robin LeBlanc

**Affiliations:** 1 School of Education, Acadia University, 22 Crowell Drive, Wolfville, NS B4P 2R6 Canada; 2 Division of Otolaryngology Head and Neck Surgery, Department of Surgery, Dalhousie University, 5850 University Avenue, Halifax, NS B3K 6R8 Canada; 3 Department of Otolaryngology Head and Neck Surgery, King Saud University, Riyadh, Saudi Arabia; 4 Department of Otolaryngology Head and Neck Surgery, Hail University, Hail, Saudi Arabia; 5 Division of Infectious Disease, Department of Internal Medicine, Dalhousie University, 5780 University Avenue, Halifax, NS B3H 1V7 Canada

**Keywords:** Thyroiditis, Thyroid abscess, Salmonella, Suppurative thyroiditis

## Abstract

**Background:**

Thyroid gland infections are rare. Their incidence is estimated to be less than 1% in immunocompromised hosts. Most common pathogens isolated are Gram positive aerobic cocci. Infections with Gram negative facultative aerobes such as *Salmonella* are rare.

**Case presentation:**

A 55-year-old female with type II diabetes mellitus and a history of a colloid right thyroid lobe nodule presented with neck pain and fever. She was found to have a thyroid abscess 2 weeks following a non-specific diarrheal illness. A needle aspiration for symptomatic and diagnostic purposes was performed. Cultures grew *Salmonella enterica serotype Heidelberg*. She was treated with a 12-week course of oral antibiotics and serial aspiration.

**Conclusion:**

A thyroid abscess is a rare occurrence; however, a high index of suspicion is required to make the diagnosis. The management is directed at minimizing morbidity. The mainstay treatment is medical, but surgery is sometimes necessary to achieve adequate source control, particularly when complications arise.

## Background

Thyroid infections are a rare entity because of the unique anatomical location and physiological characteristics the gland possesses [1, 2]. Acute Suppurative Thyroiditis (AST) is commonly seen in abnormal thyroid glands. Another predisposing factor for this condition is an immunocompromised state [3]. It is caused by bacterial pathogens, of which Gram-positive aerobes such as *Staphylococcus aureus* and *Streptococcus pyogenes* are the most common isolates [4–6]. Infections with Gram-negative facultative aerobes such as *Salmonella spp.* are rare, which could be life-threatening in immunocompromised patients.

Complications from the infection could range from recurrent laryngeal nerve injury, airway obstruction, sepsis, and death [7–9]. Therefore, prompt diagnosis and proper management can prevent such complications [10]. In this manuscript, we report on a case of a thyroid abscess due to *Salmonella spp.* in an immunocompromised patient. We also provide a retrospective review of all cases of AST due to *Salmonella spp.* reported in the English literature from January 1980 through December 2019 in the MEDLINE, EMBASE, and Scopus databases. The search terms used were thyroid abscess, suppurative thyroiditis, and salmonella.

## Case presentation

A 55-year-old woman presented to the emergency department with a chief complaint of acute onset right-sided neck pain that developed over 12–24 h. The pain was continuous and dull in nature, was felt in the right anterior neck, was non-radiating, aggravated by neck rotation, had no relieving factors, and was rated at 10/10 in severity. It was associated with a fever of 39.9 degrees Celsius measured orally, diaphoresis, and chills. She denied having any change in voice or difficulty in breathing or swallowing. Her past medical history included multiple colloid cysts in her right thyroid lobe followed by serial ultrasound (US), as well as other comorbidities such as type II diabetes mellitus (DM), hypertension, hypothyroidism, gastroesophageal reflux disease and dyslipidemia. Of note, she had dental cleaning and a nonspecific diarrheal illness for 48 h, 17 days prior to her presentation, respectively. Her past surgical history included a tonsillectomy as a child. Her social history revealed no recent travel, no bird or farm exposure, and no sick family contacts. She denied using any illicit drugs. She had no pets and was a lifelong non-smoker. Her immunization status was up to date. Her medications included spironolactone, irbesartan, sitagliptin, canagliflozin, levothyroxine, aspirin, rosuvastatin, rabeprazole, and vortioxetine. She had multiple allergies, including penicillin and sulfa drugs, which caused hives. She also reported a rash with macrolides.

**Fig. 1:**
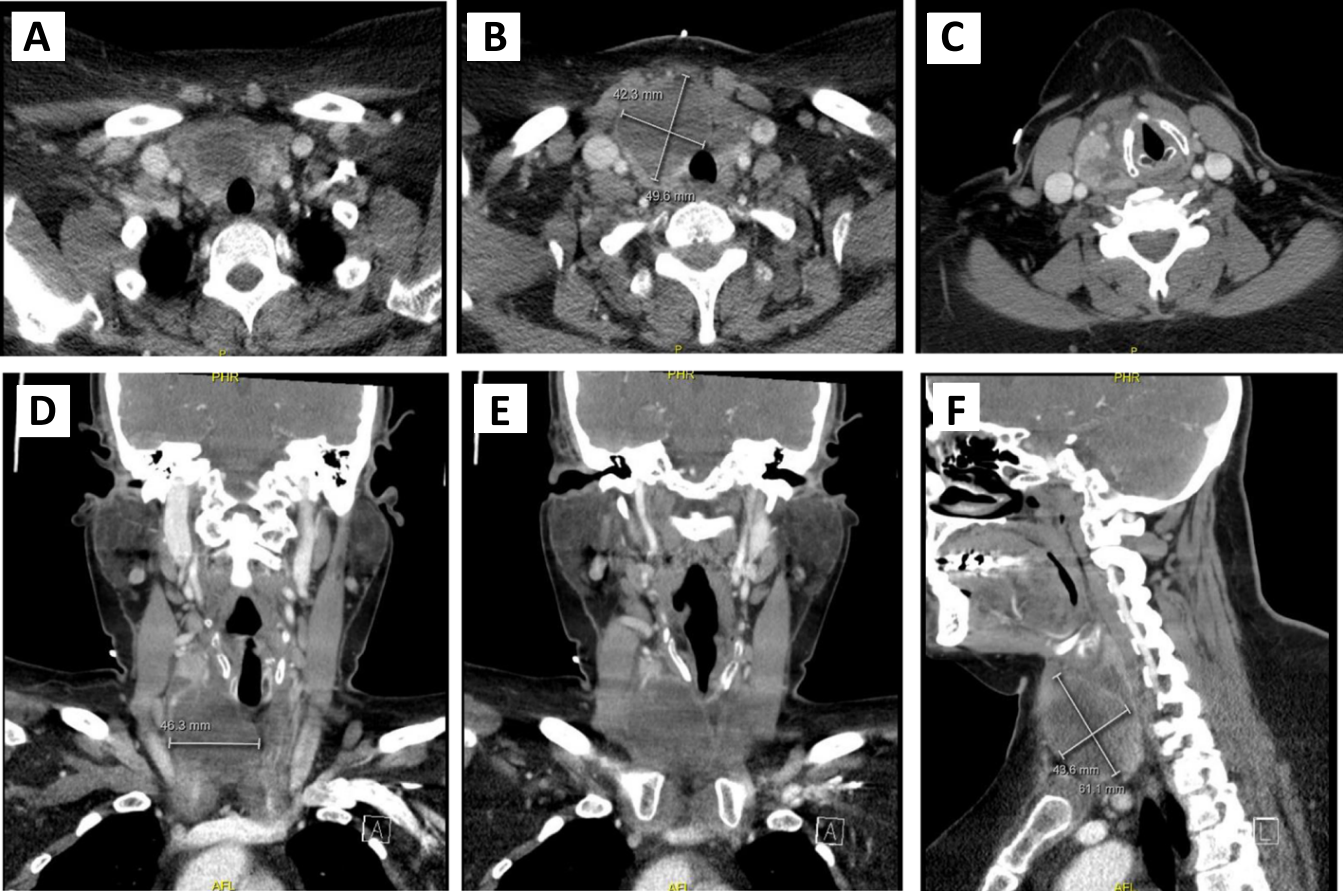
An enhanced CT scan of the neck on initial presentation. **a-c** showing the axial views from superior to inferior, **d-f** showing the coronal views from anterior to posterior and **f** showing the sagittal view. Images are showing a large lobulated cystic lesion with some thin septations, measuring about 6.1 × 4.4 × 4.6 cm in maximal craniocaudal, anterior-posterior, and transverse dimensions, respectively

**Fig. 2:**
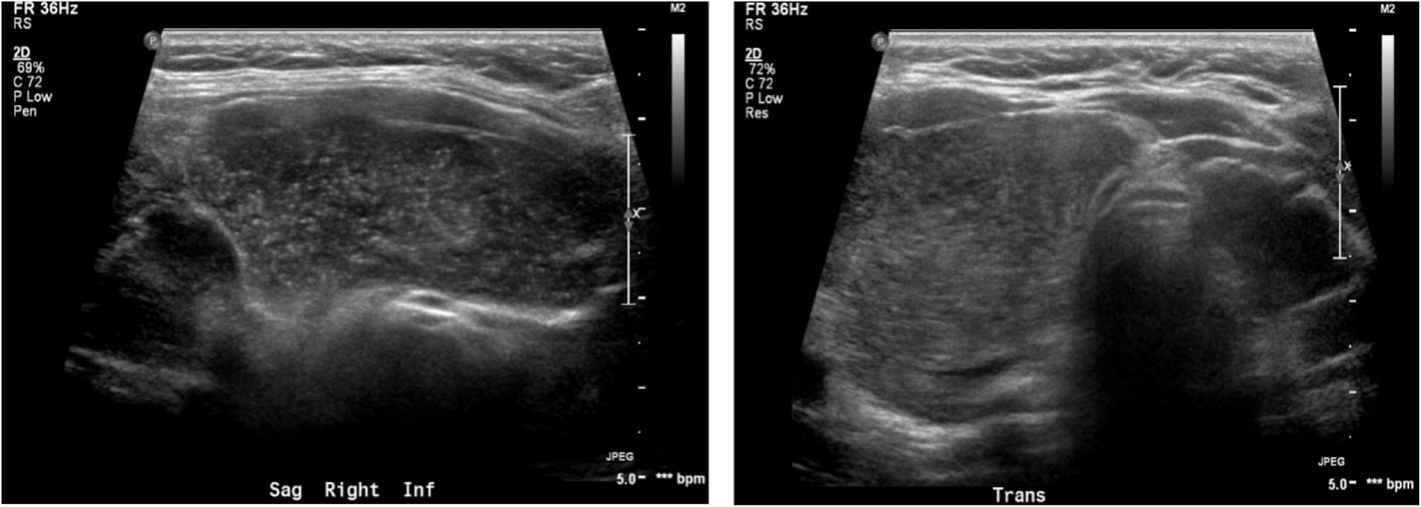
Ultrasound of the neck. Showing a large complex cystic lesion in the mid to lower portion of the right lobe. It measures about 4.9 cm × 2.3 cm × 4.8 cm

On examination, she appeared well, had no stigmata of endocarditis, and no lymphadenopathy. She did not have a hoarse voice or stridor. Examination of her ears, nose, throat and oral cavity was normal. Flexible nasal endoscopy revealed a normal looking nasopharynx, oropharynx, and hypopharynx, with normal vocal cord mobility. Inspection of her neck showed an asymmetric right-sided prominence, with overlying erythema. There was diffuse tenderness and fullness of the lower right side of the neck. There were no limitations in range of motion of the neck. A complete blood count revealed leukocytosis at 20.8 × 10 ^9^/L with a predominance of neutrophils. Blood culture and urinalysis were unremarkable. Thyroid stimulating hormone (TSH) level was 0.77mIU/L and Hemoglobin A1c (HbA1c) was 7.8%. A contrast-enhanced computed tomography (CT) scan of the neck demonstrated a large cystic lesion in the right thyroid lobe that measured 6.1 × 4.4 × 4.6 cm (cm) (Fig. 1). A correlation made with a prior surveillance US done 7 months earlier showing an increase from 4.9 × 2.3 × 4.8 cm (Fig. 2).

Following the CT scan, the patient received a 1 g dose of intravenous (IV) ceftriaxone. An initial attempt at percutaneous drainage was unsuccessful and the patient was discharged on 500 mg of cephalexin orally four times a day. The patient returned to the emergency department 3 days later with worsening symptoms and subjective difficulties in swallowing solids. Another attempt at percutaneous drainage yielded 25 ml (mL) of purulent fluid that was sent for culture. She noticed immediate relief and a significant improvement in her symptoms. She was switched to clindamycin 450 mg orally four times a day and discharged. She was brought back to clinic 3 days later for follow up, where she was found to have recurrent symptoms. Percutaneous aspiration was performed again yielding 20 mL of purulent fluid that was again sent for culture. She noticed immediate symptomatic relief. The Infectious Diseases specialists were consulted and started the patient on ciprofloxacin 500 mg orally twice a day based on the culture results from the first aspirate fluid, which grew *Salmonella enterica* serotype Heidelberg sensitive to ceftriaxone, ciprofloxacin, and trimethoprim-sulfamethoxazole, but resistant to ampicillin.

**Fig. 3:**
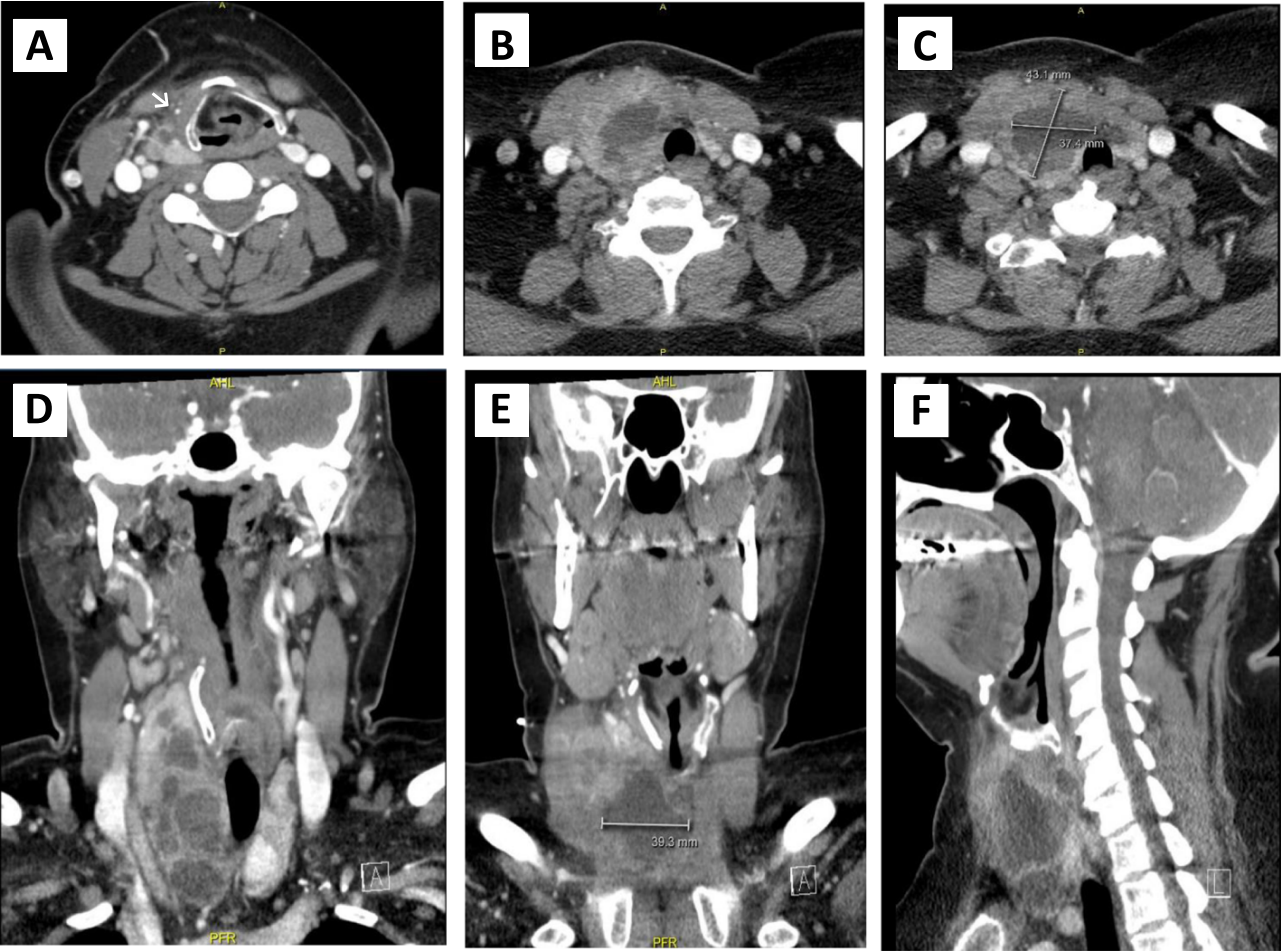
An enhanced CT scan of the neck after antibiotic therapy. **a-c** showing the axial views from superior to inferior, **d-e** showing the coronal views from anterior to posterior and **f** showing the sagittal view. The images show a loculated cystic lesion with internal septations in the mid lower lobe of the right thyroid which measures 3.7 × 4.3 × 5 cm in size. However, it has a progressively thickened heterogeneously enhancing wall. The white arrow on figure **a** pointing the right infrahyoid muscle that is slightly thickened with adjacent inflammatory stranding

She presented to the emergency department 4 days later due to recurrent symptoms. A repeat percutaneous aspiration yielded 30 mL of purulent fluid that was once again cultured. No changes were made to her antibiotics as the culture results from both subsequent aspirates were unchanged. Her white cell count dropped to 11.58 × 10 ^9^/L, and her C-Reactive Protein (CRP) was measured at 223.4 mg/L. After the third percutaneous aspiration, she continued to improve. Her white cell counts a week later had normalized, and her CRP dropped to 16.8 mg/L. A repeat contrast-enhanced CT scan a month after her initial presentation showed regression of the cystic component of the lesion to 3.7 × 4.3 × 5 cm (Fig. 3). However, the lesion had a heterogeneously rim enhancing wall and was multiseptated, with some inflammation involving the infra-hyoid strap and the sternocleidomastoid muscles (Fig. 2). Her inflammatory markers by now had normalized. Given the CT findings, a decision was made to continue with the antibiotic course for an additional 6 weeks. Two weeks following the CT scan, an US-guided aspiration yielded 3 mL of purulent fluid. However, the fluid was sterile on culture. The patient completed a total of 12 weeks on the oral ciprofloxacin and had a complete recovery, remaining symptom free at 1 year post initial presentation. Her only complication of treatment was a vaginal yeast infection, treated successfully with oral fluconazole.

## Discussion and conclusion

The thyroid gland is rarely infected due its protective fibrous capsule, rich vascularity, lymphatic drainage, and high concentration of iodine and hydrogen peroxide inhibiting bacterial growth [1, 5, 11, 12]. The incidence of AST and abscess formation is 0.1–0.7% of all reported thyroid lesions [6]. Abnormal thyroid anatomy, such as nodular goiter, cysts, and pyriform sinus fistulas can predispose to AST [5, 13, 14]. The latter originates from a third or fourth branchial cleft cyst anomaly, and can have tracts that connect the pyriform sinus and thyroid gland. Therefore, a branchial cleft cyst anomaly is a risk factor for recurrent thyroid infections and abscess formation [14]. Our patient did not have a branchial cleft anomaly but was known to have a colloid cyst in the right thyroid lobe. Interestingly, for undetermined reasons, the right lobe is more commonly involved than the left lobe in suppurative thyroiditis [15, 16].

The causative organisms of AST are mainly bacterial with few reports caused by fungi, and parasites [10]. The most common bacteria are Gram-positive aerobes such as *Staphylococcus aureus* and *Streptococcus pyogenes,* which account for 40% of cases [6]. Infections with Gram-negative aerobes account for 25%, while anaerobes account for 12% of AST cases [10]. Our review found 28 previously reported cases of S*almonella spp.* AST. Previous reports are summarized in Table 1 [1, 2, 5, 7–10, 13–34]. *Salmonella* is a motile Gram-negative anaerobic bacillus with two main species; *S. enterica* and *S. bongori* [35]. However, there are many subspecies of both. *Salmonella* thyroiditis can be caused by typhoidal *salmonella* and non-typhoidal *salmonella (NTS), with* the latter being more common [16]. Common (NTS) serotypes are *Enteriditis*, *Typhimirium*, *Newport*, *Javiana*, and *Heidelberg* [36]. NTS causes gastroenteritis in immunocompetent patients and is often non-invasive. However, a major predisposing factor to develop AST is immunocompromised status. Despite this, the incidence of thyroid infections in this population is less than 1% [3–5, 14]. Uncontrolled DM, prolonged use of steroids, Human Immunodeficiency Virus (HIV), cancer, and post-transplantation immune suppression are most commonly implicated in patients with AST secondary to *Salmonella spp.* and more specifically NTS*.* In immunocompromised patients, NTS could cause more invasive extra-intestinal infections [35, 37–39]. Our patient’s Hg A1c was elevated at 7.8% indicating suboptimal control of DM that is in keep with most cases of AST with DM in the literature [8, 10, 35].

**Table 1:** List of published cases of AST due to *Salmonella spp.*

Case Number	Publication	Number of Cases	Age& Gender	Predisposing Comorbidities	Thyroid abnormalities	***Salmonella*** Species	Intervention	
							Medical	Surgical
1	Svenungsson & Lindberg [17]	1	72 M	Steroid use	N/A	*Salmonella enteriditis*	TMP-SMX (duration not specified)	None
2	Walter and MacMonagle [18]	1	49F	None	MNG	*Salmonella choleraesuis*	Amoxicillin (duration not specified)	Thyroid lobectomy
3	Fule and Saoji [19]	1	N/A	N/A	N/A	*Salmonella* paratyphi A	N/A	N/A
4	Nmadu [20]	2	N/A	N/A	N/A	*Salmonella typhi*	N/A	N/A
5	Gudipati and Westblom [21]	1	79 M	N/A	N/A	*Salmonella* typhimurium	Ceftriaxone × 2 weeks	I&D
6	Igler, et al. [22]	1	70F	DM	MNG	*Salmonella* enteritidis	TMP-SMX × 4 weeks	I&D
7	Chiovato, et al. [23]	1	40F	None	MNG	*Salmonella* Brandenburg	Ceftriaxone × 10 days	Aspiration, I&D, and thyroid lobectomy
8	Lalitha and John [24]	2	N/A	N/A	None	*Salmonella* paratyphi A, *Salmonella cholerasius*	None	None
9	Lecuit, et al. [25]	1	48 M	HIV infection	None	*Salmonella enteriditis*	Amoxicillin × 12 days	I&D
10	Susković and Z Vucicević [26]	1	47F	DM	None	*Salmonella enteritidis*	Antibiotics (not specified)	I&D
11	Lala, et al. [27]	1	66 M	None	Thyroid nodule	*Salmonella* group D	Ciprofloxacin (duration not specified)	Subtotal thyroidectomy
12	Jasmi, et al. [14]	1	62F	None	MNG	*Salmonella typhi*	Amoxicillin-clavulinic acid × 3 days	Aspiration
13	Duraker, et al. [7]	1	52 M	DM	None	*Salmonella typhi*	Netilmicin + Clindamycin (duration not specified)	I&D
							Ofloxacin × 10 days	
14	Su and Huang [16]	1	79F	DM	MNG	*Salmonella typhimurium*	Ampicillin (duration not specified)	Thyroid lobectomy
							Ceftriaxone × 17 days	
							Ciprofloxacin (duration not specified)	
15	Dai, et al. [28]	1	82 M	CLL	MNG	*Salmonella* group B	Ceftriaxone (duration not specified)	I&D
16	Sriburee [29]	1	55F	None	MNG	*Salmonella* group C	TMP-SMX × 2 weeks	Aspiration and I&D
							Cefazolin and metronidazole (duration not specified)	
17	Chen, et al. [30]	1	60F	Invasive thymoma	MNG	*Salmonella* group D1	Ceftriaxone × 2 weeks	Thyroid lobectomy
							Oral antibiotics (duration not specified)	
18	Chou and Hsieh [31]	1	31F	None	MNG	*Salmonella choleraesuis*	Ampicillin/sulbactam	I&D
							Clindamycin and ceftriaxone (duration not specified)	
19	Krudop, et al. [32]	1	53F	None	MNG	*Salmonella* group C	Antibiotics (duration not specified)	I&D and thyroid lobectomy
20	Wu, et al. [1]	1	74 M	Renal transplant on immunosuppressive therapy	None	*Salmonella enteriditis*	Cefepime × 4 days,	Thyroid lobectomy
							Ceftriaxone × 28 days	
							Lifelong antibiotics	
21	Ambroziak, et al. [15]	1	82 M	DM, and steroid use	None	*Salmonella enteritidis*	Ceftriaxone × 2 weeks	Thyroid lobectomy
							Ampicillin × 3 weeks	
22	Kiss, et al. [2]	1	48F	HIV infection	N/A	*Salmonella* spp.	Ceftriaxone × 2 weeks	I&D
23	Kazi, et al. [33]	1	52 M	HIV infection	None	*Salmonella spp.*	Lifelong TMP-SMX	Thyroid lobectomy
24	Kuzu, et al. [9]	1	50F	DM	N/A	*Salmonella enteritidis*	Metronidazole and ceftriaxone × 5 days	I&D
							Ciprofloxacin × 4 weeks	
25	Murali & Bhandary [5]	1	26F	None	MNG	*Salmonella* Typhi	Antibiotics × 1 week (duration not specified)	Thyroid lobectomy
26	Hernik, et al. [8]	1	61F	DM	None	*Salmonella enterica*	Clindamycin, ceftazidime × 1 week	I&D
							TMP-SMX × 1 week	
27	Vengathajalam, et al. [10]	1	58F	DM	MNG	*Salmonella* spp.	Antibiotics (not specified)	Aspiration
28	Quintana, et al. [34]	1	N/A	None	N/A	*Salmonella enteriditis*	Antibiotics (not specified)	None

*F* Female, *M* Male

*N/A* Not available or not mentioned in the article, *MNG* Multinodular goiter, *DM* Diabetes mellitus, *HIV* Human Immunodeficiency Virus, *CLL* Chronic lymphocytic leukemia, *I&D* Incision and drainage, *TMP-SMX* Trimethoprim/sulfamethoxazole

*E*xtra-intestinal infection by *Salmonella* occurs by dissemination of the bacteria through the bloodstream or lymphatics [15, 40]. Haematogenous spread occurs from the gastrointestinal (GI) tract, and extra-intestinal infection ensues after distant seeding of the bacteria. *Salmonella* can also spread through the lymphatic route from the GI tract or tonsils [40]. In the majority of reported cases, a previous episode of gastrointestinal illness, upper respiratory tract infection, or pharyngitis, was implicated prior to the infection in the thyroid gland [1, 7, 10, 15–17, 22, 23, 33]. Hence, we hypothesize in our case that hematogenous spread from the GI tract during the diarrheal illness allowed for seeding of the organism in the pre-existing thyroid nodule. Furthermore, as per Telzak et al., diabetics are more prone to develop *salmonella* infections due to lower gastric acid production and slowed gastric motility [41].

Fever, chills, neck pain, lethargy, sore throat, and compressive symptoms like dysphagia and voice changes are different presentations of AST [4, 10, 15]. Thyrotoxicosis is a potential complication [9, 18, 25]. It occurs due to the release of thyroid hormones into the circulation when thyroid follicles are disrupted from the infection [14, 33, 42]. This could be detected with thyroid function tests i.e. TSH, triiodothyronine (T3), and thyroxine (T4). Our patient only had her TSH measured as a screen for thyrotoxicosis, which was normal. Potential differential diagnoses to consider for patients presenting with AST symptoms are de Quervain’s thyroiditis, medullary or anaplastic thyroid carcinoma, and subacute thyroiditis as well as other deep space neck infections [4, 16, 33, 42]. Other complications include airway obstruction, destruction of the thyroid or parathyroid glands, internal jugular vein thrombosis, recurrent laryngeal nerve injury, sepsis, and death [5, 7–9, 28]. Thus, prompt diagnosis is crucial. Blood work, imaging, and cultures are helpful in reaching the diagnosis [4, 10, 20, 24]. Blood workup includes complete blood count, inflammatory markers like CRP and erythrocyte sedimentation rate (ESR), and thyroid function tests such as TSH, T3 and T4 [4, 10, 33, 42]. Our patient had leukocytosis and elevated CRP. However, from a metabolic standpoint, TSH was within normal limits ruling out thyrotoxicosis. Imaging is very useful in reaching the diagnosis. Multiple imaging modalities can be utilised. US of the neck is a cheap, widely available, and quick tool that could be utilized for both diagnosis and therapy. Other modalities include CT scan with contrast to evaluate for deep space neck infections, and a barium swallow study to help identify the presence of a pyriform fistula [4, 10, 33, 42]. Both US and CT can help to identify extra-thyroidal extension of the infection [4, 10, 33, 42].

Treatment for AST could be medical or surgical depending on the presentation. For conservative treatment, a trial of aspiration and antibiotic administration is a reasonable initial step [1, 10, 14, 31, 42]. Similar to the case reported by Vengathajalam et al., serial aspiration and antibiotic treatment resulted in complete recovery [10]. The choice of antibiotics is dictated by the local sensitivity and resistance patterns; however, ampicillin, third generation cephalosporins, and fluoroquinolones are often appropriate first line agents. The recommended duration of therapy is a minimum of 10–14 days. However, treatment of at least 4–5 weeks is reasonable if surgery was not performed to eradicate the infection [8]. Surgical therapies can include incision and drainage, hemithyroidectomy or total thyroidectomy [14, 42]. A formal incision and drainage or more involved surgery might not be necessary in the absence of complications. Furthermore, surgery in these situations might carry a higher risk of bleeding and injury to the recurrent laryngeal nerve given the presence of inflammation and scarring in the thyroid bed. On the other hand, surgery might be necessary if there is a high suspicion of malignancy, or persistence of infection [5, 15, 16, 18, 21, 23, 27, 30, 32, 33].

To conclude, AST is a rare occurrence. One must have a high index of suspicion when a patient presents with signs and symptoms of AST. *Salmonella* has a predilection for structurally abnormal tissues, such as cystic or mixed thyroid nodules. Both immunocompetent and immunocompromised patients can develop AST. Immunocompromised patients have a more virulent clinical course and poorer outcomes, including death. The purpose of management is to minimize morbidity; thus, quick diagnosis and early treatment is crucial. The mainstay treatment is medical, but surgery may be necessary to achieve adequate source control particularly in the presence of complications.

## Data Availability

Data sharing is not applicable to this article as no datasets were generated or analysed during the current study. Data sharing is unavailable for this study as it would compromise patient privacy. However, further information regarding the case is available, within limits of patient privacy, upon request.

## Abbreviations

CT: Computed tomography
US: Ultrasonography
CRP: C-reactive protein
ESR: Erythrocyte sedimentation rate
cm: Centimeters
g: Grams
mg: Milligrams
GI: Gastrointestinal
mL: Milliliters
HgA1c: Hemoglobin A1c
AST: Acute Suppartive Thyroiditis
TSH: Thyroid Stimulating Hormone
T4: Thyroxine
T3: Triiodothyronine
NTS: Non typhoidal *salmonella*
DM: Diabetes mellitis
HIV: Human Immunodeficiency Virus
I&D: Incision and drainage
TMP-SMX: Trimethoprim sulfamethoxazole
CLL: Chronic lymphocytic leukemia
